# The Preventive Effect of Melatonin on Radiation-Induced Oral Mucositis

**DOI:** 10.3390/cells12172178

**Published:** 2023-08-30

**Authors:** Reiko Tokuyama-Toda, Hirochika Umeki, Mitsuru Okubo, Chika Terada-Ito, Toshio Yudo, Shinji Ide, Susumu Tadokoro, Masashi Shimozuma, Kazuhito Satomura

**Affiliations:** Department of Oral Medicine and Stomatology, School of Dental Medicine, Tsurumi University, 2-1-3, Tsurumi, Tsurumi-ku, Yokohama City 230-8501, Japan; umekingdem@icloud.com (H.U.); flying-arcriner0326@outlook.jp (M.O.); terada-chika@tsurumi-u.ac.jp (C.T.-I.); ut2015mail27integral@gmail.com (T.Y.); ide-shinji@tsurumi-u.ac.jp (S.I.); tadokorosusumu@yahoo.co.jp (S.T.); masashi.shimozma@gmail.com (M.S.); satomura-k@tsurumi-u.ac.jp (K.S.)

**Keywords:** free radical scavenger, melatonin, oral mucosa, radiation-induced oral mucositis

## Abstract

Melatonin exerts various physiological effects through melatonin receptors and their ability to scavenge free radicals. Radiotherapy is a common treatment for head and neck tumors, but stomatitis, a side effect affecting irradiated oral mucosa, can impact treatment outcomes. This study investigated the preventive effect of melatonin, a potent free radical scavenger, on radiation-induced oral mucositis. Mice were irradiated with 15 Gy of X-ray radiation to the head and neck, and the oral mucosa was histologically compared between a melatonin-administered group and a control group. The results showed that radiation-induced oral mucositis was suppressed in mice administered melatonin before and after irradiation. It was suggested that the mechanism involved the inhibition of apoptosis and the inhibition of DNA damage. From these findings, we confirmed that melatonin has a protective effect against radiation-induced oral mucositis**.**

## 1. Introduction

Melatonin, an amino acid derivative with a molecular weight of 232.28, is primarily produced and secreted from the pineal gland [1]. It is synthesized from tryptophan through stepwise enzymatic modifications involving serotonin [2,3,4]. The physiological effects of melatonin encompass circadian rhythm control [5], hypnotic action [6], body temperature regulation [6], antigonadal [7], sexual cycle control [8], immunostimulatory [9], antioxidant [10,11,12], anti-inflammatory [13,14], and antitumor [15,16] effects. These actions are mediated through melatonin receptors present in cell membranes, which are expressed in numerous tissues and cells throughout the body [17,18]. The antioxidant action of melatonin is mainly targeted at mitochondria [19,20]. In addition, melatonin possesses the ability to scavenge free radicals within its own molecules. It can directly neutralize various free radicals, including hydroxyl radicals, and indirectly activate antioxidative enzymes within the body [21,22,23,24,25]. Notably, melatonin exhibits high efficacy in neutralizing the highly toxic hydroxyl radical (●OH) [26]. These direct and/or indirect properties make melatonin potentially valuable as a radioprotective and anticancer agent [27]. Administration of melatonin has been shown to improve the survival rate of mice exposed to lethal doses of ionizing radiation [27,28] and to protect bone marrow cells and lymphocytes [29,30,31,32].

Oral cancer ranks as the 16th most common cancer globally [33]. Despite the decline in its incidence worldwide, countries with high rates of tobacco use, alcohol consumption, and an aging population have an increasing number of oral cancer cases [33,34]. The overall five-year survival rate for oral cancer is approximately 50%, which is lower than that of other cancers, largely due to late-stage detection [35]. Given these circumstances, chemotherapy and radiation therapy are often employed alongside surgery for oral cancer treatment. Radiotherapy, in particular, is valuable as an adjuvant treatment after surgery and for advanced cases. However, as the oral region falls within the radiation field, the development of acute radiation injury, specifically oral mucositis, is inevitable [36,37]. This often necessitates interruptions or suspensions in irradiation, although continuous radiation therapy is known to be more effective. Radiation-induced oral mucositis significantly impacts the patient’s quality of life and treatment outcomes. Therefore, preventing radiation-induced oral mucositis in conjunction with radiotherapy for oral cancer is crucial [38,39,40].

In this study, we focused on the previously reported physiological action of melatonin as a free radical scavenger, and its efficacy as a prophylactic agent for radiation-induced oral mucositis was examined.

## 2. Materials and Methods

This study involved male ICR mice and received approval from the Animal Experiment Committee of Nihon University under the approval numbers AP11D004 (approval date: 1 August 2011) and AP13D015-1 (approval date: 6 December 2013). All methods were conducted in compliance with relevant guidelines and regulations. Surgical procedures were performed with sodium pentobarbital anesthesia, and measures were taken to minimize the animals’ suffering. Thirty male ICR mice, aged 6 weeks, were obtained from CLEA Japan, Inc. (Tokyo, Japan). They were housed under a 12 h light–dark cycle at 22 °C and fed a normal diet.

### 2.1. X-ray Irradiation

First, the potential preventive effect of melatonin administration against radiation-induced oral mucosal damage in mice was investigated. The mice were divided into three groups: a control group (*n* = 10), an irradiation group (IR group, *n* = 20), and an irradiation group with melatonin administration (IR + Mel group, *n* = 10). The experimental protocol involved the following: 1. Control group: Mice were maintained under normal conditions throughout the study period, and tissue samples were collected. 2. IR + Mel group: After an initial breeding period, the mice were intraperitoneally administered 100 mg/kg of melatonin (Sigma Aldrich, Darmstadt, Germany) on the day of irradiation. Thirty minutes later, the head and neck were exposed to 15 Gy X-ray radiation. Subsequently, the mice were provided with water containing 50 μg/mL of melatonin and a normal diet. Tissue samples were collected on the seventh day. 3. IR group: After an initial breeding period, the mice in this group received an intraperitoneal administration of a vehicle on the day of irradiation. Thirty minutes later, the head and neck were exposed to X-ray radiation at a dose of 15 Gy. The mice were then provided with normal water and a normal diet. Tissue samples were collected on the seventh day.

### 2.2. Mouse Tissue Samples

The tongue and buccal mucosa of each group of mice were extirpated, fixed with 4% paraformaldehyde (Wako Pure Chemical Industries Ltd., Osaka, Japan), and embedded in paraffin. Then, 5 μm thick sections were cut, deparaffinized, and stained with hematoxylin and eosin. All sections were mounted and observed under a microscope. Furthermore, to quantitatively compare morphological changes in tissue, the length of the epithelial leg in the tongue and the number of nucleated cells observed in the epithelium in the buccal mucosa were used as indicators to compare the three groups. Ten fields of interest (550 × 750 μm) for each group (*n* = 5) were randomly selected. The length of the epithelial leg in the tongue and the number of nucleated cells in the buccal mucosa were calculated.

### 2.3. TDT-Mediated dUTP End-Labeling (TUNEL) Staining

The numbers of apoptotic cells in tongue tissues in the three groups were detected by TUNEL staining using an In Situ Cell Death Detection Kit, Fluorescein (Roche, Mannheim, Germany). Following the manufacturer’s instructions, all cells showed blue nuclear DAPI staining, but TUNEL-positive cells displayed green nuclear staining. The stained sections were analyzed using laser-scanning confocal microscopy. Ten fields of interest (730 × 500 μm) for each group (*n* = 5) were randomly selected, and the number of TUNEL-positive cells was counted.

### 2.4. Immunohistochemical Staining

The tongue sections from the three groups underwent immunohistochemistry to detect the expression of cleaved caspase-3, high mobility group box 1 (HMGB1), and 8-hydroxy-2′-deoxyguanosine (8-OHdG). The primary antibodies used in this study were as follows: anti-cleaved caspase-3 antibody (rabbit) (Cell Signaling Technology, Inc., Danvers, MA, USA) at a 1:400 dilution in phosphate-buffered saline (PBS, pH 7.4) with 1% bovine serum albumin (BSA), anti-HMGB1 antibody (rabbit) (Merck, Darmstadt, Germany) at a 1:200 dilution in PBS (pH 7.4) with 1% BSA, and anti-8-OHdG antibody (rabbit) (Bioss, Inc., Boston, MA, USA) at a 1:500 dilution in PBS (pH 7.4) with 1% BSA. For the secondary antibody, antirabbit IgG (goat) conjugated with Texas Red^®^ (Abcam, Cambridge, UK) was used at a 1:1000 dilution in PBS (pH 7.4) with 1% BSA. After deparaffinization following the standard procedure, endogenous peroxidase was blocked by treating the sections with 3% H_2_O_2_ in methanol for 1 h at room temperature (RT). The sections were then treated with 10% normal goat serum at RT for 10 min, followed by overnight incubation with the respective primary antibody at 4 °C. Subsequently, the sections were washed with PBS and incubated with the secondary antibody for 1 h at RT. After washing with PBS, the cell nuclei were stained with DAPI (Merck) for 10 min at RT. Finally, the slides were mounted in glycerol (ZEISS, Oberkochen, Germany) and analyzed using laser-scanning confocal microscopy. The specificity of the immunoreaction was confirmed by incubating with normal rabbit IgG or normal rabbit serum instead of the primary antibody. For each molecule, 10 randomly selected fields of interest measuring 730 × 500 μm for each group (*n* = 5) were counted to determine the number of cleaved caspase-3-positive, HMGB1-positive, and 8-OHdG-positive cells.

### 2.5. Cell Culture

RT7 cells, an oral mucosal epithelial cell line derived from human buccal mucosa [41], was a generous gift from Dr. Nobuyuki Kamata, Hiroshima University, Hiroshima, Japan. RT7 cells were cultured in a KGM-Gold Basal Medium (Lonza Ltd., Basel, Switzerland) supplemented with 0.4% bovine pituitary extract, 0.1% recombinant human epidermal growth factor, 0.1% bovine insulin, 0.1% hydrocortisone, 0.1% transferrin, 0.05% epinephrine, 0.1% gentamicin, and 0.1% amphotericin-B. The culture was maintained at 37 °C in a humidified atmosphere with 5% CO_2_, and the medium was changed twice a week.

### 2.6. Cell Proliferation after Irradiation

The effect of irradiation on the proliferation of RT7 cells was analyzed using a crystal violet staining method [42]. In this study, we investigated the impact of melatonin on RT7 cell proliferation and further examined its effect after irradiation. First, the cells were seeded at a density of 4 × 10^3^ cells/well in 12-well culture plates. In the control group, the cells were treated with various concentrations (0, 100, 500, and 1000 μM) of melatonin for different durations ranging from 1 to 17 days. In the irradiated group, the cells were treated with the same melatonin concentrations for 30 min, followed by irradiation with 10 Gy of X-ray radiation. After irradiation, the cells were cultured with the respective melatonin concentrations (*n* = 4). For both groups, the cells were rinsed with PBS on scheduled days and fixed with 1% glutaraldehyde in PBS overnight at 4 °C. Subsequently, the cells were stained with 0.02% crystal violet in deionized water for 30 min at RT. After several rinses with distilled water, the crystal violet bound to the cells was extracted by incubating with 500 μL/well of 70% ethanol overnight at 4 °C. The absorbance was measured at 570 nm using a microplate reader.

### 2.7. Statistical Analysis

All data were analyzed using Scheffe’s test; *p* < 0.05 and *p* < 0.01 was considered statistically significant (SPSS, Inc., Chicago, IL, USA).

## 3. Results

### 3.1. Histological Examination of Oral Tissues (Tongue and Buccal Mucosa) after Irradiation with or without Melatonin Administration

**Figure 1:**
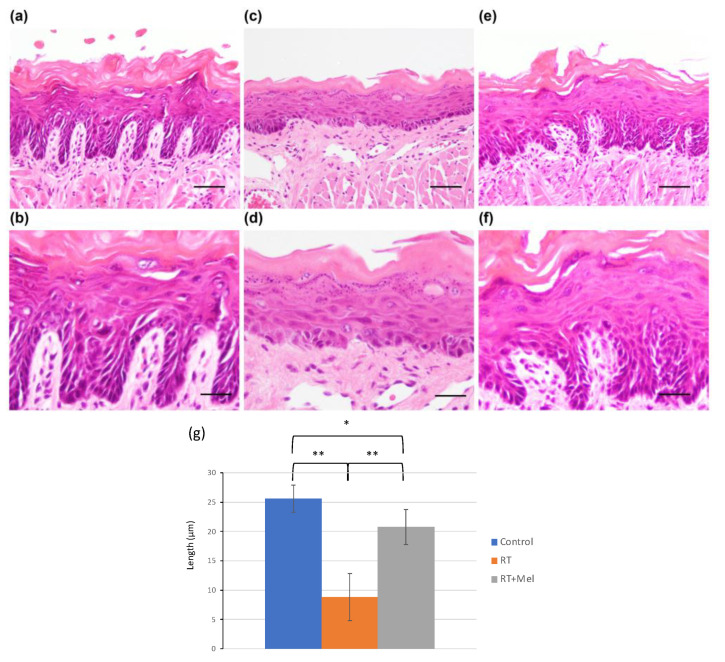
Histology of the tongue in each group. (**a**,**b**) Control group (no irradiation). (**c**,**d**) IR group (vehicle intraperitoneal administration + 15 Gy irradiation + normal water feeding). (**e**,**f**) IR + Mel group (intraperitoneal administration of melatonin + 15 Gy irradiation + melatonin-containing water breeding). H–E staining. The basal layer exhibited cell enlargement, dense nuclei, an increased nucleus-to-cytoplasm ratio, and disordered arrangement. In addition, polygonal cells, degenerated cells, and keratohyalin-like granules were observed sporadically. The entire epithelium showed atrophy, dilation of capillaries in the lamina propria, and edematous space in the tongue muscle. In contrast, the tongues of mice in the IR + Mel group exhibited partial radiation damage. However, the thickness of the epithelial layer was maintained, and only slight disturbance was observed in the basal layer. Compared with the IR group, radiation damage was suppressed in the IR + Mel group, and the histological images resembled those of the nonirradiated control group. (**a**,**c**,**e**) Bars are 50 μm. (**b**,**d**,**f**) Bars are 20 μm. (**g**) Graph of the length of the epithelial leg in the tongue. * *p* < 0.05, ** *p* < 0.01. In the IR group, the epithelial leg flattened significantly compared with the control group, whereas in the IR + Mel group, the flattening of the epithelial leg was suppressed, and the length was maintained.

**Figure 2:**
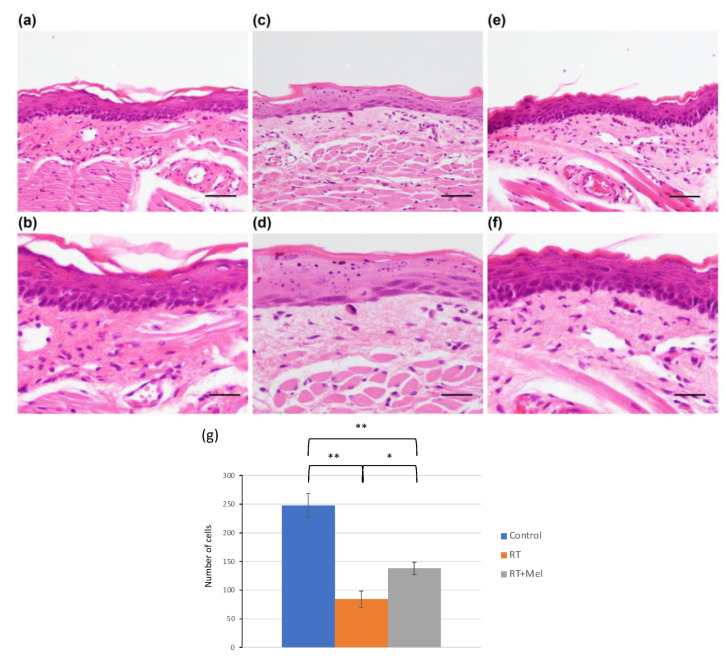
Histology of the buccal mucosa in each group. (**a**,**b**) Control group (no irradiation). (**c**,**d**) IR group (vehicle intraperitoneal administration + 15 Gy irradiation + normal water feeding). (**e**,**f**) IR + Mel group (intraperitoneal administration of melatonin + 15 Gy irradiation + melatonin-containing water breeding). H–E staining. As in the tongue, radiation damage was observed in both the epithelium and lamina propria in the IR group, whereas radiation damage was suppressed in the IR + Mel group. Histological images similar to those in the control group were observed. (**a**,**c**,**e**) Bars are 50 μm. (**b**,**d**,**f**) Bars are 20 μm. (**g**) Graph of the number of nucleated cells observed in the epithelium in the buccal mucosa. * *p* < 0.05, ** *p* < 0.01. The IR group showed a significant decrease in cell number compared to the control group. In contrast, the IR + Mel group suppressed the decrease in cell number and maintained the number of nucleated cells.

We first examined the histology of the oral mucosa in two groups: the IR + Mel group, which received melatonin before and after irradiation; and the IR group, which received a vehicle before irradiation. The goal was to investigate whether melatonin administration could prevent radiation damage to the oral mucosa. In the tongues of mice in the IR group, we observed ambiguous intercellular bridges in the epithelium, along with cell enlargement, darkening of the nucleus, increased nucleocytoplasmic (N/C) ratio, and disordered arrangement in the basal layer. In addition, scattered polygonal cells, degenerated cells, and keratohyalin-like granules were noted. The entire epithelium showed atrophy, and there was dilatation of capillaries and edema in the tongue muscle space in the lamina propria (Figure 1c,d). These findings are consistent with radiation-induced oral mucositis. In contrast, in the tongues of mice in the IR + Mel group, we observed partial radiation-induced oral mucositis. The thickness of the epithelial layer was maintained, with a slight disturbance in the basal layer (Figure 1e,f). Compared with the IR group, radiation-induced oral mucositis was suppressed in the IR + Mel group, with a histology more similar to that of the nonirradiated control group (Figure 1a,b). Then, to quantitatively compare these histological changes among the three groups, a graph was drawn using the length of the epithelial leg as an indicator. As a result, in the IR group, the epithelial legs were observed to flatten significantly compared with the control group, whereas, in the IR + Mel group, the flattening of the epithelial legs was significantly suppressed (Figure 1g). In addition, a similar histopathology was observed in the examination of the buccal mucosa. In the IR group, radiation-induced oral mucositis was observed in both the epithelium and lamina propria (Figure 2c,d), whereas it was suppressed in the IR + Mel group (Figure 2e,f). Tissue images in the IR + Mel group resembled those of the control group (Figure 2a,b). To quantitatively compare these histological changes among the three groups, the number of nucleated cells observed in the epithelium in the buccal mucosa was counted in each group. As a result, the IR group showed a significant decrease in cell number compared to the control group, whereas, in the IR + Mel group, the decrease in the number of nucleated cells was suppressed (Figure 2g). These findings suggest that the administration of melatonin before and after irradiation can suppress radiation-induced oral mucositis.

### 3.2. Investigation of the Mechanism of the Effect of Melatonin in Suppressing Radiation-Induced Oral Mucositis (TUNEL Staining)

**Figure 3:**
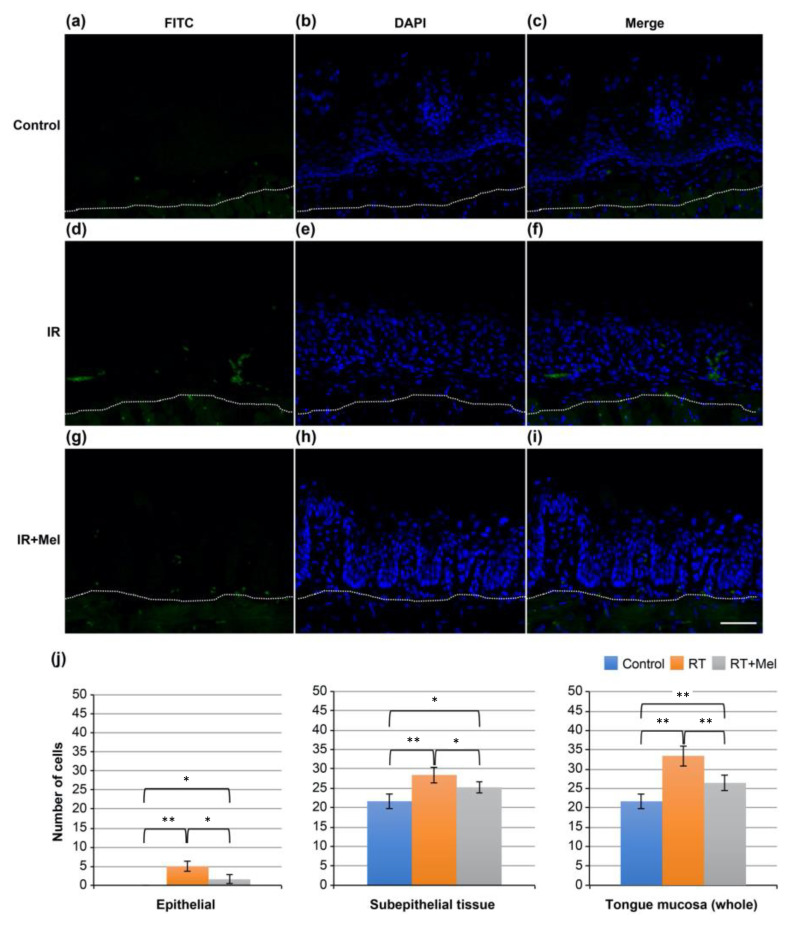
TUNEL staining image in the tongue. (**a**–**c**) Control group. (**d**–**f**) IR group. (**g**–**i**) IR + Mel group. (**a**,**d**,**g**) FITC. (**b**,**e**,**h**) DAPI. (**c**,**f**,**i**) merge. The scale bar is 50 μm. All images have the same magnification. The white dashed line represents the boundary between the epithelium and subepithelial tissue. (**j**) Graph of the number of TUNEL-positive cells in the epithelium, subepithelial tissue, and full thickness of the tongue mucosa. * *p* < 0.05, ** *p* < 0.01. Significantly, more positive cells were observed in the IR group than in the control group in all regions of the epithelium, subepithelial tissue, and mucosa. On the other hand, the number of positive cells in the RT + Mel group was higher than that in the control group, but it was significantly lower than that in the IR group.

Then, the mechanism underlying the preventive effect of melatonin administration on radiation-induced oral mucositis was investigated, focusing on apoptosis as one of the representative phenomena induced by irradiation. TUNEL staining was performed on tongue tissues from the control group, IR group, and IR + Mel group. FITC-positive cells, indicating TUNEL-positive cells, were observed in all groups (Figure 3a–i). Subsequently, ten fields were randomly selected in each group, and the number of positive cells was separately counted in the epithelium, subepithelial tissue, and whole mucosa. A comparison was made among the three groups. The results showed a significantly higher number of positive cells in the IR group compared with the control group in all regions of the epithelium, subepithelial tissue, and whole mucosa. On the other hand, in the IR + Mel group, the number of positive cells was higher than in the control group, but significantly lower than in the IR group (Figure 3j). These findings suggest that melatonin administration may suppress radiation-induced oral mucositis by inhibiting radiation-induced apoptosis.

### 3.3. Investigation of the Mechanism of the Effect of Melatonin in Suppressing Radiation-Induced Oral Mucositis (Cleaved Caspase-3)

**Figure 4:**
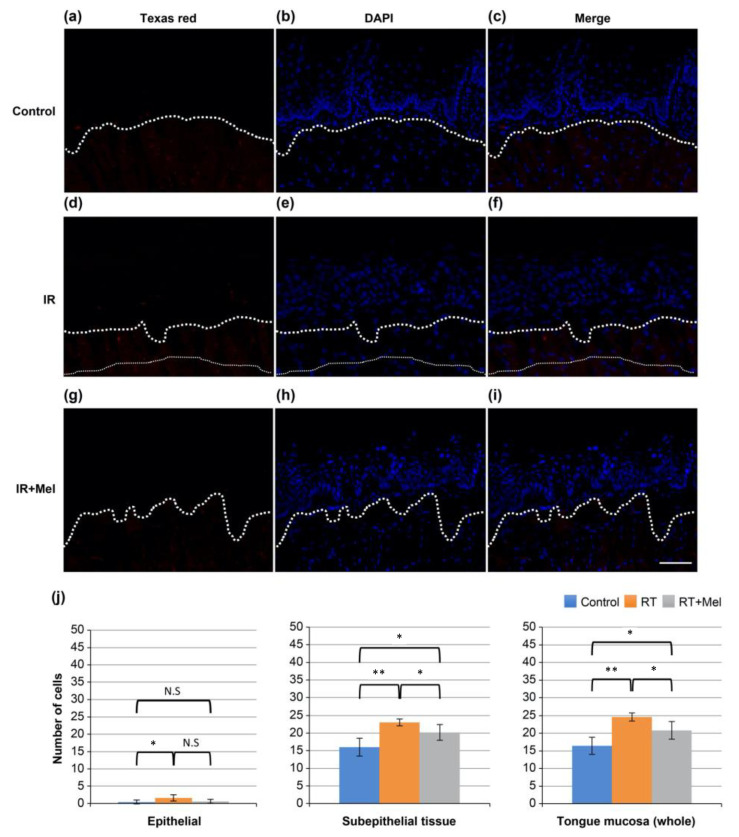
Immunohistochemical staining image of active caspase-3 in the tongue. (**a**–**c**) Control group. (**d**–**f**) IR group. (**g**–**i**) IR + Mel group. (**a**,**d**,**g**) Texas red for cleaved caspase-3. (**b**,**e**,**h**) DAPI. (**c**,**f**,**i**) merge. Scale bar is 50 μm. All images have the same magnification. The white dashed line represents the boundary between the epithelium and subepithelial tissue. (**j**) Graph of the number of cleaved caspase-3-positive cells in the epithelium, subepithelial tissue, and full thickness of the tongue mucosa. * *p* < 0.05, ** *p* < 0.01. Significantly more positive cells were observed in the IR group than in the control group in all regions of the epithelium, subepithelial tissue, and mucosa. On the other hand, although the RT + Mel group had more positive cells than the control group, it tended to decrease compared with the IR group.

In addition, we investigated the activation of caspase-3, which is a molecular mechanism involved in cell modification and apoptosis induction following irradiation. Immunohistochemical analysis was performed on tissues from the three groups mentioned earlier. Texas red-positive cells, indicating cleaved caspase-3-positive cells, were observed in all groups (Figure 4a–i). Subsequently, ten fields were randomly selected in each group, and the number of positive cells was counted in the epithelium, subepithelial tissue, and whole mucosa. A comparison was made among the three groups. The results showed significantly more positive cells in the IR group compared with the control group in all regions of the epithelium, subepithelial tissue, and whole mucosa. On the other hand, the IR + Mel group had more positive cells than the control group, but there was a tendency toward a decrease compared with the IR group (Figure 4j). These findings suggest that melatonin administration may suppress radiation-induced apoptosis by inhibiting caspase-3 activation.

### 3.4. Investigation of the Mechanism of the Effect of Melatonin in Suppressing Radiation-Induced Oral Mucositis (HMGB1)

**Figure 5:**
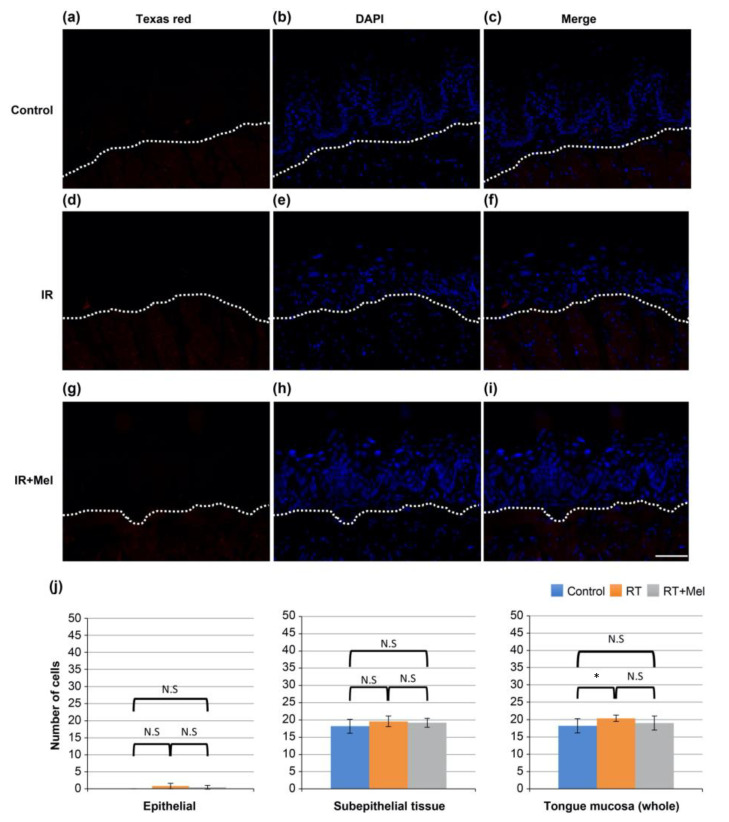
Immunohistochemical staining image of HMGB1 in the tongue. (**a**–**c**) Control group. (**d**–**f**) IR group. (**g**–**i**) IR + Mel group. (**a**,**d**,**g**) Texas red for HMGB1. (**b**,**e**,**h**) DAPI. (**c**,**f**,**i**) merge. The scale bar is 50 μm. All images have the same magnification. The white dashed line represents the boundary between the epithelium and subepithelial tissue. (**j**) Graph of the number of HMGB1-positive cells in the epithelium, subepithelial tissue, and full thickness of the tongue mucosa. * *p* < 0.05. Although there was a significant difference between the control group and IR group in the whole mucosa, there was no significant difference between the other groups.

In addition, we focused on HMGB1, which is an indicator of necrotic cells, and immunohistochemically examined the tissues of each of the three groups in the same manner as in the previous section. As a result, Texas red-positive cells; that is, HMGB1-positive cells, were observed in each group (Figure 5a–i). Then, ten fields were randomly selected in each group, and the number of positive cells was separately counted in the epithelium, subepithelial tissue, and whole mucosa and compared among the three groups. As a result, there was a significant difference between the control group and the IR group in the whole mucosa, but there was no significant difference between the other groups (Figure 5j). Based on the above results, melatonin administration had no inhibitory effect on the number of necrotic cells indexed with HMGB1.

### 3.5. Investigation of the Mechanism of the Effect of Melatonin in Suppressing Radiation-Induced Oral Mucositis (8-OHdG)

**Figure 6:**
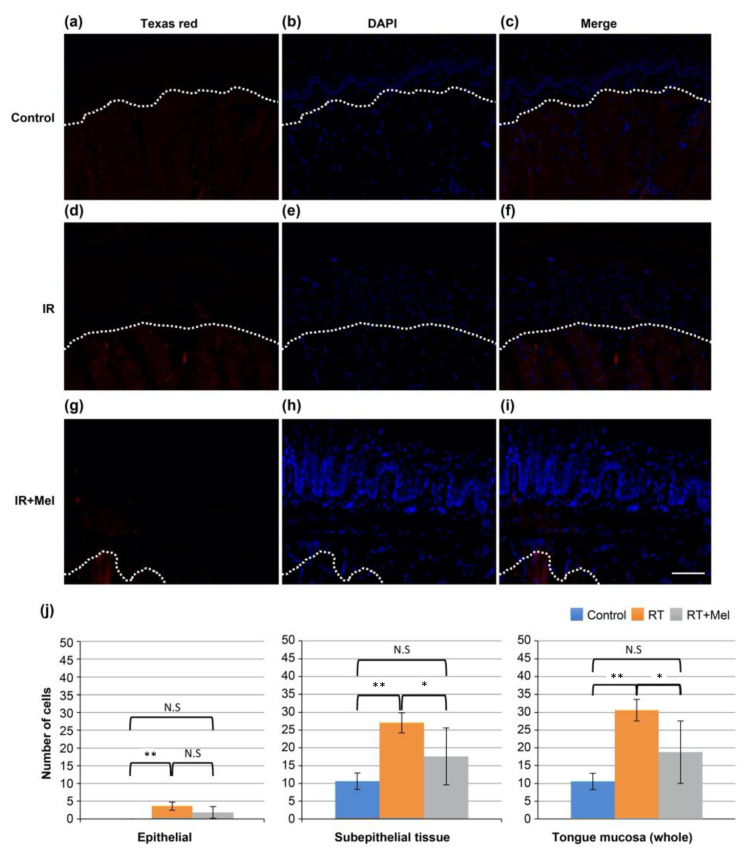
Immunohistochemical staining image of 8-OHdG in the tongue. (**a**–**c**) Control group. (**d**–**f**) IR group. (**g**–**i**) IR + Mel group. (**a**,**d**,**g**) Texas red for 8-OHdG. (**b**,**e**,**h**) DAPI. (**c**,**f**,**i**) merge. Scale bar is 50 μm. All images have the same magnification. The white dashed line represents the boundary between the epithelium and subepithelial tissue. (**j**) Graph of the number of 8-OHdG-positive cells in the epithelium, subepithelial tissue, and full thickness of the tongue mucosa. * *p* < 0.05, ** *p* < 0.01. Significantly more positive cells were observed in the IR group than in the control group in all regions of the epithelium, subepithelial tissue, and mucosa. On the other hand, it was significantly decreased in the RT + Mel group compared with that in the IR group. The number of positive cells tended to be higher than in the control group, but the difference was not significant.

**Figure 7:**
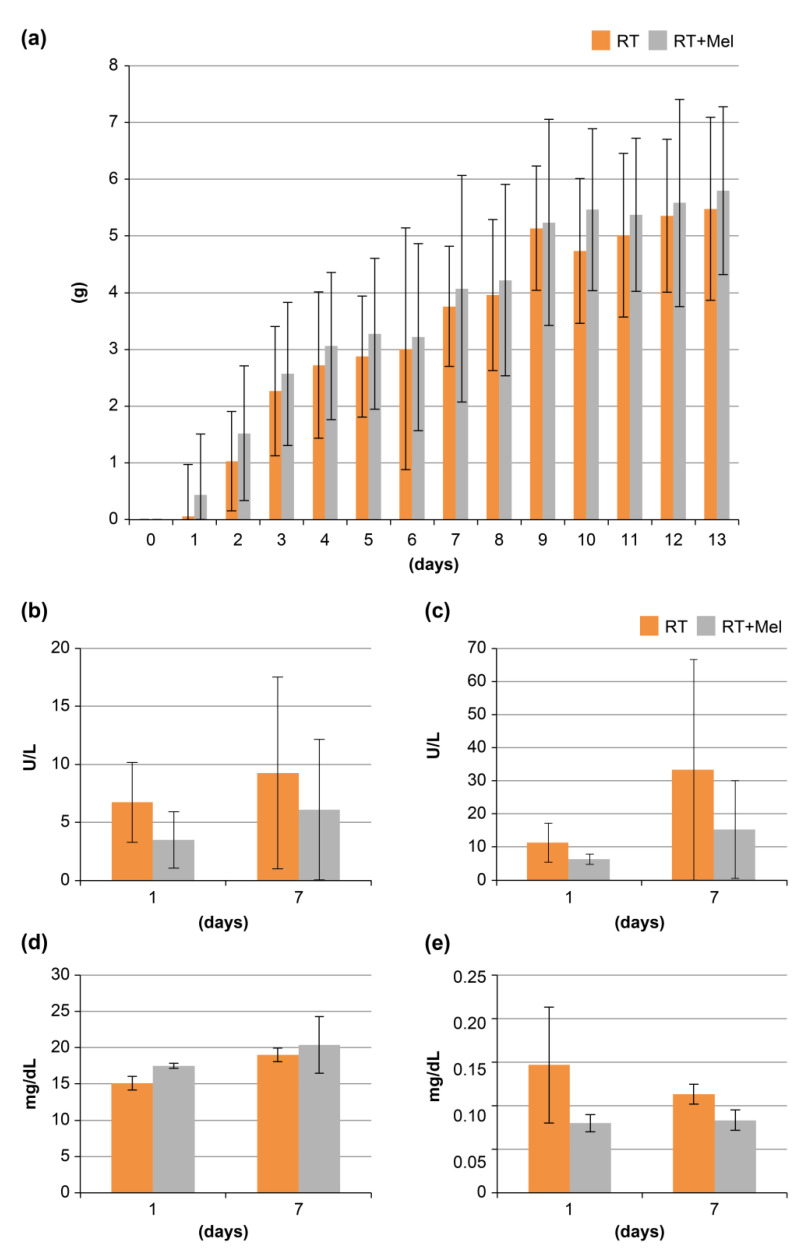
Side effects due to melatonin administration. (**a**) Weight gain. (**b**–**e**) Peripheral blood biochemistry test results. (**b**) AST. (**c**) ALT. (**d**) BUN. (**e**) Creatinine. Changes in body weight (A) and serum levels of AST (B), ALT (C), BUN (D), and creatinine (E) in the experimental period. Generally, none of these laboratory parameters were significantly affected by melatonin application throughout the experimental period. Values are mean ± SE from 10 animals per group.

Furthermore, we investigated 8-OHdG, an indicator of necrotic cells, by performing immunohistochemical analysis on tissues from each of the three groups, following the same procedure as in the previous section. Texas red-positive cells, indicating HMGB1-positive cells, were observed in all groups (Figure 6a–i). Subsequently, ten fields were randomly selected in each group, and the number of positive cells was independently counted in the epithelium, subepithelial tissue, and whole mucosa. A comparison was made among the three groups. The results showed a significant difference only between the control group and the IR group in the whole mucosa, whereas there was no significant difference between the other groups (Figure 6j). Based on these findings, melatonin administration did not have an inhibitory effect on the number of necrotic cells indicated by HMGB1. Meanwhile, BW and levels of AST, ALT, BUN, or creatinine were not affected by melatonin administration throughout the whole experimental period, suggesting that melatonin administration caused no adverse effects (Figure 7).

### 3.6. Cell Biological Study of Oral Mucosal Epithelial Cells after Irradiation with or without Addition of Melatonin

**Figure 8:**
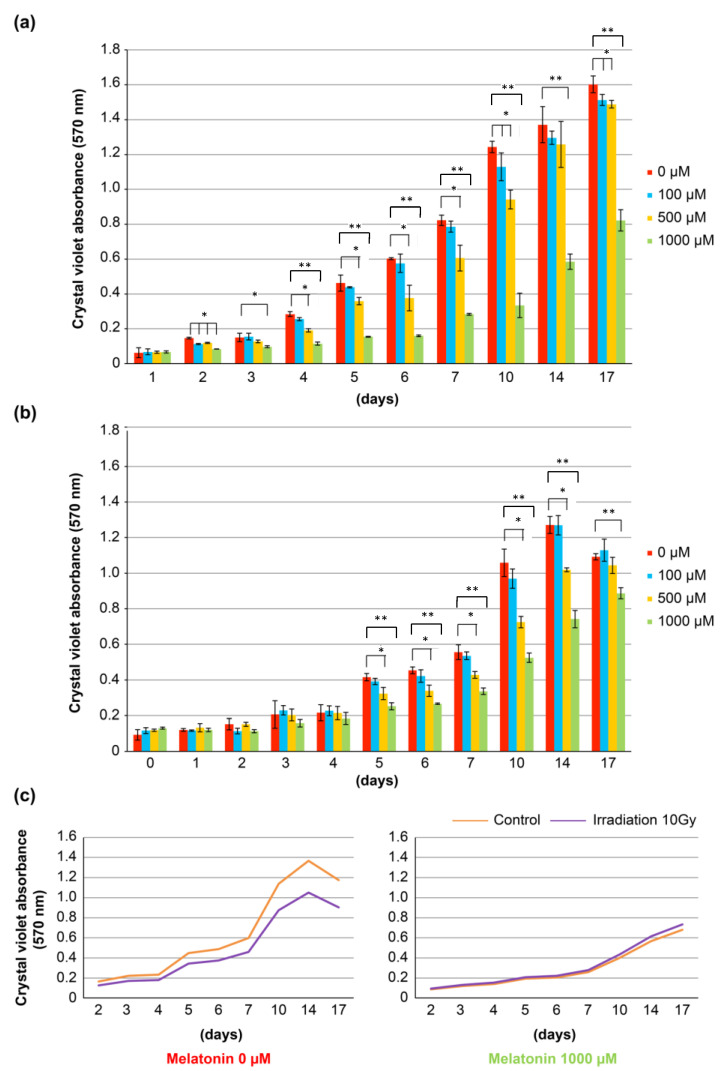
Effect of melatonin on the proliferation of RT7 cells. (**a**) Effect of the addition of various melatonin concentrations on cell proliferation in a normal culture sample. * *p* < 0.05, ** *p* < 0.01. (**b**) Effect of various melatonin concentrations on the proliferation of RT7 cells after irradiation with X-ray 10 Gy. * *p* < 0.05, ** *p* < 0.01. (**c**) Effects of addition and non-addition of melatonin on cell proliferation with and without X-ray irradiation. The transition of cell proliferation is shown in the graph. It was revealed that melatonin has a cytostatic effect on RT7 cells under normal culture conditions. After irradiation, RT7 cells were influenced by irradiation, so the proliferation was slower than that in a normal culture sample, but cell proliferation was suppressed depending on the melatonin concentration. Among these results, the effect of melatonin was examined in nonX-ray irradiation and 10 Gy irradiation. In the absence of melatonin, cell proliferation was suppressed in the irradiation 10 Gy group compared with the control group, but when 1000 μM melatonin was added, there was no significant difference in proliferation between the control group and irradiation 10 Gy group.

Then, the effect of melatonin on the proliferation of human oral mucosal epithelial RT7 cells was examined after in vitro irradiation. To understand the effect of melatonin on RT7 cells under normal conditions, we added melatonin to normal culture samples and assessed cell proliferation before the irradiation examination. The results revealed that melatonin has a cytostatic effect on RT7 cells under normal conditions (Figure 8a). Based on this finding, we added various concentrations of melatonin 30 min before irradiation and evaluated the growth of RT7 cells after exposure to 10 Gy. The results showed that cell proliferation was suppressed in a melatonin concentration-dependent manner, although the overall proliferation was slower than in the normal culture samples due to the influence of irradiation (Figure 8b). To assess the direct effect of melatonin, we compared changes in cell growth with and without the addition of melatonin in two groups: nonX-ray irradiation and 10 Gy irradiation. The results indicated that cell proliferation was suppressed in the 10 Gy irradiation group compared with the control group without melatonin addition. However, in the melatonin 1000 μM addition group, there was no significant difference in proliferation between the control group and the 10 Gy irradiation group, suggesting that the addition of melatonin alleviates the decrease in cell proliferation caused by radiation damage (Figure 8c).

## 4. Discussion

Melatonin has been reported to have various physiological effects on multiple organs [43]. These effects include sleep rhythm restoration by oral intake of melatonin [44,45,46], reduction in insulin resistance associated with experimental type 2 diabetes [47], and prevention of mitochondrial dysfunction, as well as antiapoptotic effects in experimental ischemic stroke [48,49], cardioprotective action [50,51,52], improvement of implantation rate and pregnancy rate in pregnancy [53], and reduction in chronic kidney disease and liver fibrosis [54,55,56]. Previous studies have also shown that melatonin promotes bone formation, is produced in the salivary glands, and has a physiological role in tooth development [57,58,59,60]. In this study, we focused on the fact that melatonin is a versatile protective agent against oxidative DNA damage and hypothesized that it could reduce damage to normal oral mucosa by suppressing oxidative stress associated with radiotherapy for oral cancer [19,20,61]. In addition, melatonin has been reported to have suppressive effects on various stages of cancer development, progression, and metastasis, suggesting that it may not interfere with other cancer treatments being conducted simultaneously [62].

To investigate the oral mucosal damage associated with radiotherapy for oral cancer, mice were divided into two groups: the IR group (without melatonin administration) and the IR + Mel group (with melatonin administration). In the IR + Mel group, melatonin was administered before and after X-ray irradiation of 15 Gy to the head and neck of the mice. Histological examination revealed various abnormalities in the tongue and buccal mucosa of the IR group compared with the nonirradiated mice (control group), indicating the occurrence of radiation-induced oral mucositis. However, in the IR + Mel group, the histological damages in the tongue and buccal mucosa were reduced, and the histological images resembled those of the control group. In the tongue, the epithelial leg development is histologically characteristic, the length of that was used as an indicator, and in the buccal mucosa, the number of nucleated cells was used as an indicator for quantitative comparison. As a result, statistically significant differences were observed. This indicated that melatonin administration could suppress radiation-induced oral mucositis. Further investigation into the mechanism revealed that melatonin suppresses apoptosis by inhibiting caspase-3 activation and DNA damage. These effects were considered part of the mechanism underlying the suppression of radiation-induced oral mucositis. In addition, melatonin was found to suppress the radiation-induced reduction in cell proliferation in oral mucosal epithelial cells, suggesting that melatonin may protect normal cells and mitigate radiation-induced damage. However, melatonin has a growth-inhibitory effect on RT7 cells under normal culture conditions, and the effect of irradiation on cell growth may depend on the cell cycle. Therefore, further detailed investigations are necessary in the future. Furthermore, this study did not examine the effect of melatonin administration on cell necrosis. It will be necessary to explore factors other than HMGB1 to confirm the effects of melatonin on cell necrosis and investigate alternative pathways.

On the other hand, the antioxidant activity of melatonin has been reported to correlate with its anti-inflammatory activity [13,14,63]. Although this study focused on the antioxidant effect of melatonin, it is necessary to investigate the preventive mechanism of radiation-induced oral mucositis from the viewpoint of its anti-inflammatory effect. Furthermore, it has been reported that melatonin upregulates indoleamine-2,3-dioxygenase 1 (IDO1) levels [64,65,66]. Melatonin is synthesized enzymatically from tryptophan when metabolized along the serotonin pathway [67]. Various kynurenine metabolites are synthesized when tryptophan is metabolized along the kynurenine pathway [68,69,70]. IDO1 is the initial rate-limiting enzyme of this pathway [71,72]. These two pathways of tryptophan metabolism have been implicated in regulating several immune and nerve diseases [71,73]. It has previously been reported that melatonin administration upregulates the expression of IDO1 [64]. Furthermore, a recent report shows that low NO activates IDO1 [74]. In this study, the upregulation of IDO1 by administered melatonin may have been related to the mechanism of suppression of radiation-induced oral mucositis. To clarify this, it is necessary to examine the effects of melatonin on tryptophan metabolism from various signaling pathways [75,76,77] as well as the expression of NOS. In this study, it was revealed histologically that the administration of melatonin suppressed radiation-induced oral mucositis. However, the details of this mechanism are still unclear. In the near future, it will be necessary to conduct further studies from the multiple perspectives mentioned above.

This study demonstrated that melatonin has the potential to suppress damage to the oral mucosa associated with radiotherapy. In the treatment of oral cancer, acute radiation-induced mucositis is common, which often leads to treatment interruptions or discontinuation due to the inclusion of normal oral mucosa within the radiation field. Prevention of radiation-induced oral mucositis, which greatly impacts treatment adherence, is crucial for the success of oral cancer treatment. Melatonin, which is already widely used for sleep disorders and known to have minimal side effects, presents an opportunity to effectively prevent radiation-induced oral mucositis [78,79,80]. Melatonin is a safe molecule available over the counter in the United States and commonly prescribed in European countries. If it can be safely administrated and if it can effectively prevent radiation-induced oral mucositis, it would be a significant contribution to oral cancer treatment.

Numerous studies have already demonstrated that melatonin slows the progression of various cancer types [81,82,83,84,85,86], including breast cancer, ovarian cancer, leiomyosarcoma, pancreatic cancer, liver cancer, colon cancer, and lung cancer [87,88,89,90,91,92,93,94]. Extensive research has explored its mechanisms of action, and studies aiming for clinical applications have been reported [95]. Although studies on oral cancer are limited, some reports suggest its potential antitumor effects [96,97,98]. Based on the findings of this study, further investigations are warranted to determine the clinical application of melatonin as a preventive agent for radiation-induced oral mucositis. The next step involves administering melatonin during radiotherapy to mice with cancer, evaluating the effects of radiation therapy and the antitumor effects of melatonin. Simultaneously, detailed examinations of the preventive effects of radiation-induced oral mucositis on normal mucosa are necessary. In addition, it is crucial to determine whether melatonin inhibits the effects of radiotherapy on cancer cells, the original purpose of the treatment. It is necessary to establish whether melatonin can prevent radiation-induced oral mucositis in normal mucosa while also exhibiting therapeutic effects against cancer without interfering with the original intent of radiation therapy. In the near future, we will conduct these studies and strive for the clinical application of melatonin as a preventive agent for radiation-induced oral mucositis.

## 5. Conclusions

In this study, it was confirmed that melatonin has a protective effect against radiation-induced oral mucositis. It was suggested that this effect may be expressed with the direct and/or indirect inhibition of apoptosis, DNA damage, and cytoprotective effect.

## Data Availability

The data that support the findings of this study are available from the corresponding author upon reasonable request.
